# Neonatal Plasmacytoid Dendritic Cells (pDCs) Display Subset Variation but Can Elicit Potent Anti-Viral Innate Responses

**DOI:** 10.1371/journal.pone.0052003

**Published:** 2013-01-10

**Authors:** Xiaoming Zhang, Alice Lepelley, Elie Azria, Pierre Lebon, Gwenaelle Roguet, Olivier Schwartz, Odile Launay, Claude Leclerc, Richard Lo-Man

**Affiliations:** 1 Régulation Immunitaire et Vaccinologie, Institut Pasteur, Paris, France; 2 INSERM U1041, Paris, France; 3 Unit of Innate Defense and Immune Modulation, Key Laboratory of Molecular Virology and Immunology, Institut Pasteur of Shanghai, Chinese Academy of Sciences, Shanghai, China; 4 Virus et Immunité, Institut Pasteur, Paris, France; 5 URA CNRS 3015, Paris, France; 6 Department of Obstetrics and Gynecology, Groupe Hospitalier Bichat Claude Bernard, Paris, France; 7 Paris 7 Diderot University, Paris, France; 8 Assistance Publique–Hôpitaux de Paris, Hopital Cochin-Saint Vincent de Paul, EA1833 et Virologie, Paris, France; 9 Assistance Publique–Hôpitaux de Paris, Hôpital Cochin, CIC-BT505, Paris, France; 10 Université Paris Descartes, Faculté de Médecine, Paris, France; Centre d'Immunologie de Marseille-Luminy, CNRS-Inserm, France

## Abstract

Neonates are highly susceptible to infectious diseases and defective antiviral pDC immune responses have been proposed to contribute to this phenomenon. Isolated cord blood pDCs innately responded to a variety of TLR7 and TLR9 dependent viruses, including influenza A virus (IAV), human immunodeficiency virus (HIV) or herpes-simplex virus (HSV) by efficiently producing IFN-α, TNF-α as well as chemokines. Interestingly, following activation by CpGA, but not viruses, cord pDCs tend to survive less efficiently. We found that a hallmark of pDCs in neonates is an extended CD2+pDCs compartment compared to adult pDCs without affecting the antiviral IFN-α response. Within CD2+pDCs, we identified a subpopulation expressing CD5 and responsible for IL-12p40 production, however this population is significantly decreased in cord blood compared to adult blood. Therefore, neonatal pDCs clearly display variation in phenotype and subset composition, but without major consequences for their antiviral responses.

## Introduction

How the immune system in early infancy accommodates pathogens is poorly defined and requires assessment to contrive and improve treatments and vaccines [1]. Newborns are considered to have defective innate immunity, possibly due to reduced inflammatory responses of innate cells to Toll-like receptor (TLR) activation [2] and limited or impaired antigen-presenting cell functions [3]. Studies showed an age-dependent maturation of the IL-12/IFN–γ response [4], [5]. In humans, cell intrinsic epigenetic regulation has been shown to impair the capacity of cord monocyte derived DCs (MoDCs) to produce IL-12 in response to LPS [6]. MoDCs also showed a limited IL-12 response to cytomegalovirus (CMV), although efficient T cell responses develop upon congenital CMV infection [7]. Exceptions exist with TLR7/8 agonists potently activating monocytes for TNF-α production [8].

Viral diseases are a major cause of mortality and morbidity in young children [1], and pDCs potently produce antiviral IFN-α in response to viruses mainly via two major innate recognition receptors of viral nucleic acid, TLR7 and TLR9 [9]. Neonatal murine studies indicate that the dendritic cell compartment including pDCs in lymphoid organs is not intrinsically impaired for innate and antigen-presenting cell (APC) functions [10], [11], [12]. However, pDCs isolated from cord blood have been shown to be defective for CpGA/TLR9 activation due to a limited IRF7 nuclear translocation [13], [14]. Fine tuning of different DCs signals vary depending on the pathogen [15], we thus reinvestigated this issue by extending the study of pDC activation by RNA viruses recognized through TLR7, including IAV [16] and HIV [17] and we further analyzed pDC subsets and phenotypes in neonates.

## Materials and Methods

### Blood and ethics statement

Heparinized cord blood samples processed within 24 h were collected from healthy full-term neonates (gestation time from 37 to 41 weeks) from Maternity Port Royal and Bichat, both in Paris area, between 2007 and 2010. Buffy coats from 18–60 year-old donors were obtained from the EFS. This study was carried out with the approval of Institut Pasteur Review Board qualified for Medical research and the French Regional Ethics Committee CPP IDF IV in agreement with the principles of the declaration of Helsinki. Written consent was obtained from the mother. Ethnic origin of cord blood donors was not available, but as a reference the geographical origins of children born in 2009 in the maternity was as follows : 48% from Europe, 14% from North Africa, 19% from Sub-Saharan Africa, 5% from Asia. Ethnic origin of adult donors was not available, and all blood samples were collected in Paris area.

### Media and reagents

Medium was RPMI-1640 with 5–10% fetal calf serum (ICN Biomedicals, Inc.), 5×10^−5^ M of 2-Mercaptoethanol (Sigma), and antibiotics (penicillin 100 U/ml, streptomycin 100 µg/ml, GIBCO BRL). TLR9 agonists CpGA (2216, 5′-GGGGACGATCGTCGGGGGG-3′) was synthesized by Sigma. Influenza virus strain A/PR8/1976 (H1N1) was from Charles River Laboratories and used as IAV (live) or HI-IAV (heat-inactivated). Stocks of HSV-1 (Shealy) were prepared from supernatants of infected Vero cells cultured in RPMI–2% FCS for 72 h p.i. at an m.o.i. of 0.1 and had a titer of 10^6^ to 10^7^ PFU/ml. For HIV, primary cells were cocultured with infected MT4C5 T cells (10^5^/ml). HI-IAV was obtained by treating the virus at 56°C for 1 hr. IL-3 and GMCSF were from R&D. Antibodies against CD1c, CD2, CD5, CD14, CD20, CD40, CD45RA, CD86, CD123 and HLADR were from eBioscience, BDCA2 and IFN-α from Miltenyi Biotec, IL-12 p40 from BD Biosciences.

### Cell purification and analysis

Mononuclear cells (MNC) were separated from granulocytes, erythrocytes, and platelets by gradient fractionation (Lymphoprep Axis-Shield). pDCs were isolated from CBMCs (cord blood mononuclear cells) or adult PBMCs (peripheral blood mononuclear cells) by using anti–BDCA-4 magnetic beads (Miltenyi Biotec) according to the manufacture's protocol. In short, 100 µl magnetic beads were used for 10^8^ cells in a volume of 500 µl buffer (PBS containing 1% fetal calf serum, 2.5 mM EDTA). After incubation at 4°C for 15min, cells were washed and run on autoMACS Pro (Miltenyi Biotec) by using possels program). Enriched pDCs were further sorted as CD123^hi^CD45RA^+^ by flow cytometry on FACSAria (BD Biosciences). In general, 2–3×10^5^ and 5–10×10^5^ pDCs were sorted from one cord blood and one buffy coat, respectively. In some experiments, pDCs were further sorted as CD2^+^ and CD2^−^. Purified pDCs and subsets were routinely more than 99% pure.

To measure the frequency of pDCs, pDC subsets or their phenotypes, 10^6^ to 3×10^6^ CBMCs or PBMCs were stained and analyzed. Cells were stained with optimally diluted fluorochrome-conjugated antibodies at room temperature for 10 minutes. Then the cells were acquired on CyAn (Beckman Coulter) and analyzed by Flowjo software (Tree Star) with computed compensation. At least 10^4^ pDCs were gated for analysis.

To measure the survival rate of stimulated pDC, purified pDCs stimulated for 24 hrs were washed twice with PBS and stained with Annexin V Apoptosis detection Kit (BD Biosciences) according to the manufacture's instructions. Cells were acquired on CyAn and analyzed as above.

### Innate activation and Cytokine Measurement

Fresh WB, 5×10^5^ MNC or 10^4^ purified pDCs were plated in 96-well flat-bottom plates (WB and MNC in a volume of 250 µl) or U-bottom plates (pDCs in a volume of 200 µl) for stimulation. Plasma or supernatants were collected at 24 hrs and kept frozen at −80°C until detection. IFN-α, TNF-α, CCL3 and CCL4 were measured by a Multiplex kit (Luminex assay, Biosource) according to the manufacture's instructions. Briefly, appropriately diluted samples were distributed into 96-well luminex plate and incubated with fluorescent beads which were coated with first antibody recognizing individual cytokine or chemokine for 1 hr at room temperature with shaking (500 RPM). After washing 3 times, secondary biotin antibodies were added and the plate was further incubated at room temperature for 1 hr with shaking (500 RPM). After washing 3 times, streptavidin phycoerythrin was added and incubated for 10 min at room temperature. The plate was washed for another 3 times and the beads were resuspended in running buffer and acquired on a Luminex ×100 machine by using the Starstation software (Applied Cytometry).

### Intracellular cytokine analyses

MNCs were stimulated as indicated for 6 hrs, GolgiPlug (BD Biosciences, 1∶1000 dilution) was added in the culture for the last 3 hrs. Cells were harvested, fixed and permeabilized by using the Cytofix/Cytoperm kit (BD Biosciences). Briefly, cells were first fixed in fixation/permeabilization solution for 20 min at 4°C. Following 2 wahsing steps with the permeabilization/washing solution, cells were stained with optimal concentrations of fluorochrome-conjugated antibodies against IFN-α or IL-12 p40 in the permeabilization/washing solution for 30 min at 4°C. After washing 3 times, cells were resuspended in PBS containing 1% fetal calf serum and acquired on CyAn (Beckman Coulter) and analyzed by Flowjo software (Tree Star). At leat 10^4^ targeted cells (for instance, at lease 10^4^ pDCs were acquried from MNCs if pDCs were the target cells.) were acquired for analysis.

### Statistical analysis

Unpaired *t* test was used for comparison of two groups of data presented as mean value ± SD. *P* values of <0.05 were considered statistically significant.

## Results and Discussion

**Figure 1:**
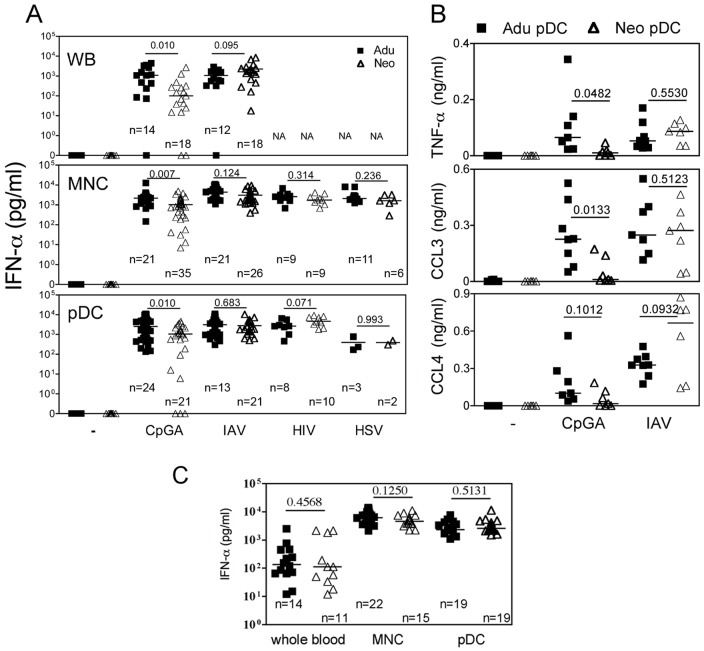
Innate responses of cord blood pDCs to viruses. Adult or neonatal whole blood (WB) (A and C), mononuclear cells (MNC) (A and C) or purified pDCs (A–C) were stimulated with CpGA (50 µg/ml for WB, 5 µg/ml for others in A), live IAV (1000 HAU/ml for WB, 10 HAU/ml for others), heat-inactivated IAV (HI-IAV in C) live HIV (10^5^ infected T cells in A) and live HSV-1 (10^6^ pfu/ml in A) for 24 hrs. Plasma or supernatants were collected and tested for IFN-α (A and C) or TNF-α, CCL3 and CCL4 (B). NA: non applicable. The number of donors is indicated for each group (n) as well as *P* values for adults and neonates comparison. In B, n = 8 for adult and n = 7 for neonates.

**Figure 2:**
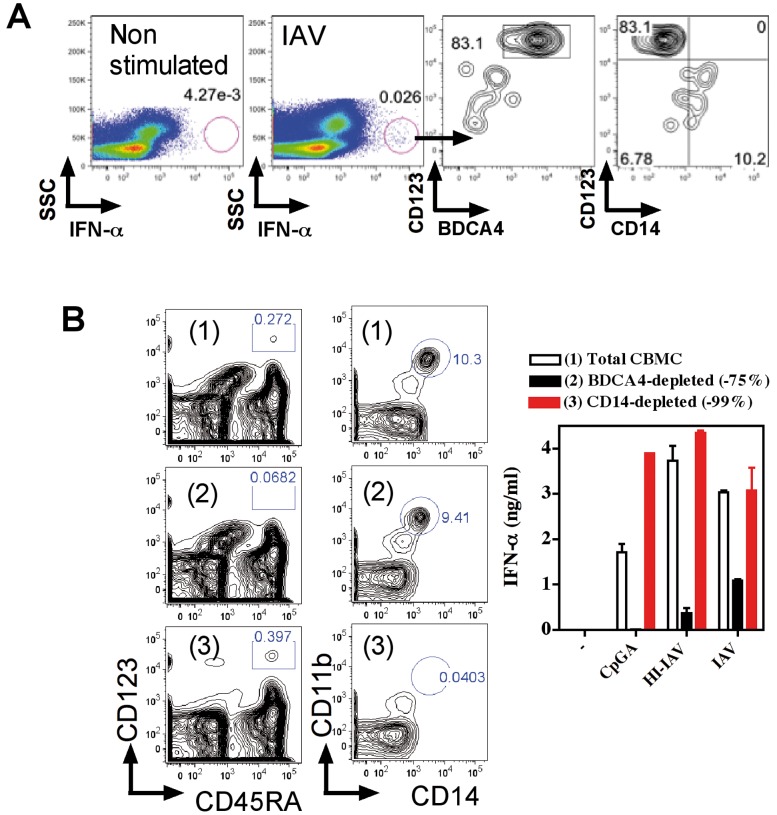
Cord blood pDCs are the main contributor for IFN-α response. (A) Neonatal cord blood was left untreated or exposed to IAV, for 6 hrs and stained intracellularly for IFN-α. pDCs are identified by CD123 and BDCA4 and monocytes by CD14 expression. (B) Total CBMC (1), or CBMC depleted of pDCs (2) or of monocytes (3) were stimulated with CpGA, live IAV and HI-IAV as in Fig. Plasma or supernatants were collected and tested for IFN-α (A). Depletion efficiency is shown for pDCs identified by CD123 and CD45RA and monocytes by CD14 and CD11b expression. One experiment representative of three is shown.

**Figure 3:**
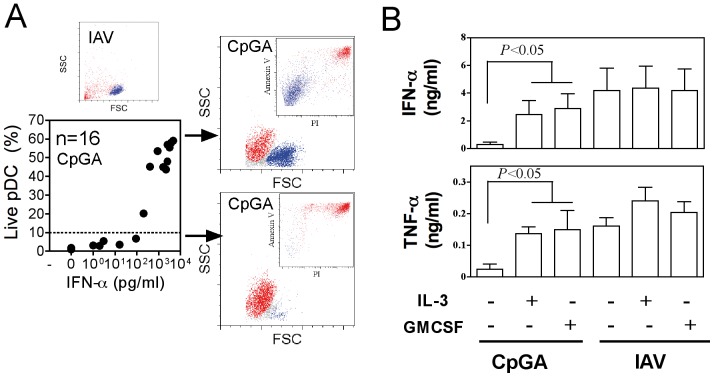
Survival fate of neonatal pDC following CpG activation. (A) IFN-α responses of neonatal pDCs to CpGA were plotted against the survival rate. Cell survival is shown by annexin V and propidium iodide staining (PI). (B) Neonatal pDCs were stimulated with CpGA or IAV in the absence or presence of IL-3 (10 ng/ml) or GMCSF (10 ng/ml) for 24 hrs. IFN-α was detected from supernatants.

We first sought to analyze the antiviral IFN-α response of cord blood cells to TLR7 dependent IAV [16] and HIV [17] as well as in response to TLR9 dependent HSV [18] and CpGA activation (Fig. 1). High levels of IFN-α secretion were observed in all conditions for neonatal whole blood and CBMC. No statistical differences were observed between neonatal and adult samples for all virus stimulation, but CpG/TLR9 displayed discrepancies. Neonatal pDCs were shown to be defective for IFN-α response to CpG/TLR9 activation, but these early studies were performed with pDCs of 50% purity [14]. pDCs can be clearly identified by the expression of BDCA4 and BDCA2, as well as the high expression of CD45RA and CD123. Therefore we further investigated functions of cord blood pDCs to >99% purity by magnetic bead enrichement of BDCA4 cells followed by FACS sorting on CD123 and CD45RA expression (Fig. S1) and then exposed these cells to viruses. pDCs purified from adult and neonatal blood responded similarly (Fig. 1A). These IFN-α responses along with the production of TNF-α and chemokines were as efficient as those obtained with adult samples, except for CpGA/TLR9 activation (Fig. 1B). We observed similar IFN-α responses to heat inactivated IAV in adult and neonatal samples tested as WB, MNC and purified pDCs (Fig. 1C). For neonatal whole blood exposed to live IAV, pDCs were mainly responsible for IFN-α secretion, whereas monocyte only poorly contributed to this response (Fig. 2A). Similarly, following depletion of pDCs, but not ofmonocytes, neonatal MNC were strongly impaired or decreased in their capacity to produce IFN-α in response to CpGA, live and HI- IAV (Fig. 2B). Interestingly, all cord blood samples responded to IAV or HIV, whereas several samples were unresponsive to CpGA as observed with both WB and isolated pDC stimulation. Indeed, we observed a lack of cell survival in several neonatal pDCs samples treated with CpGA that was not observed following viral stimulation (Fig. 3A). Non responders and low responders to CpGA for IFN-α (<100 pg/ml) was associated with poor cell survival (Fig. 3A). This phenomenon may come from the defective signaling observed by others [13]. In line with this, cord blood pDC may require stronger signaling to survive which CpGA stimulation cannot fully provide, although this was only observed in about 15% of samples. Any correlation could be made with the mode of birth delivery (natural delivery vs. caesarian), the gestational age at birth, or the sex of the neonate (Fig. S2). Alternatively, some unknown factor(s) related to development may render neonatal pDCs slightly more vulnerable to apoptosis. The underlying mechanism awaits further investigation. Cell survival as well as IFN-α and TNF-α secretion (Fig. 2B) were restored by adding IL-3 [19] or GM-CSF [20] as survival factors. In contrast to previous reports [18], we failed to detect unresponsive pDCs during HSV/TLR9 stimulation, as both neonatal MNC and purified pDCs produced IFN-α to HSV-1 stimulation (Fig. 1A) indicating that the unresponsive phenotype is not associated with a defective TLR9 pathway in cord pDCs. Our results are supported by an earlier report in which both cord blood and fetal liver pDCs responded normally to HSV stimulation by producing high amount of IFN-α [21]. In addition, this report also provided strong evidence that at earlier developmental stage pDCs expressing CD34 already have the capacity to produce IFN-α in response to HSV [21].

**Figure 4:**
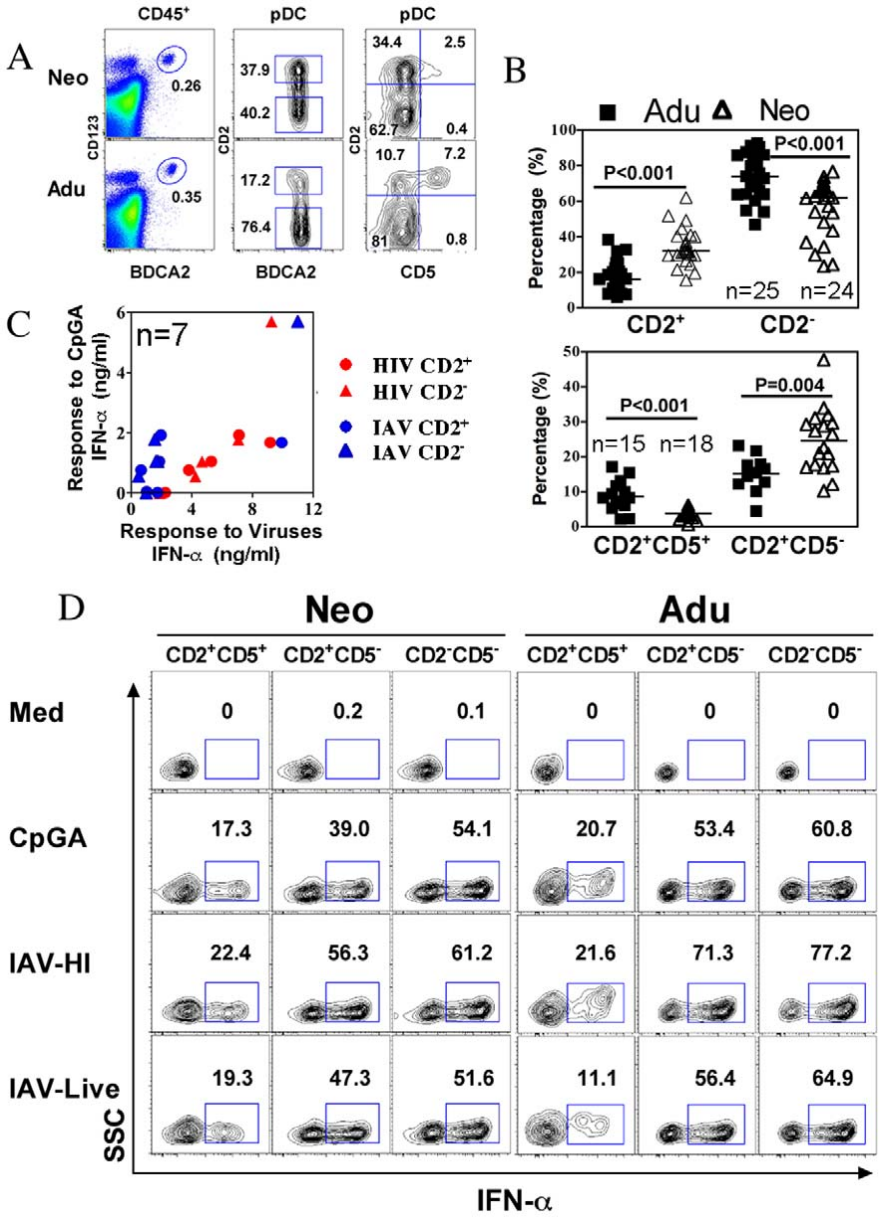
Heterogeneity of neonatal pDCs. (A) The percentages CD123^hi^BDCA2^+^ pDCs within CD45+ MNC were calculated from neonatal cord blood and adults. pDCs could be subdivided by CD2 and CD5, and the accumulated data for CD2^+^, CD2^-^ pDCs subsets and CD2^+^CD5^+^, CD2^+^CD5^−^ pDCs subsets are shown in (B). (C) The innate IFN-α production of sorted CD2^+^ and CD2^−^ pDCs subsets to CpGA, live IAV and HIV (the results are incated as ng/ml). (D) Neonatal and Adult MNCs were stimulated as indicated and CD2^+^CD5^+^, CD2^+^CD5^−^ and CD2^−^CD5^−^ pDCs subsets were gated to analyze their capacity to produce IFN-α detected by intracellular cytokine staining. Numbers indicate the percentage of cytokine-producing cells among gated cells. Data are representative of 5 experiments. The number of donors is indicated for each group (n) as well as *P* values for adults and neonates comparison.

**Figure 5:**
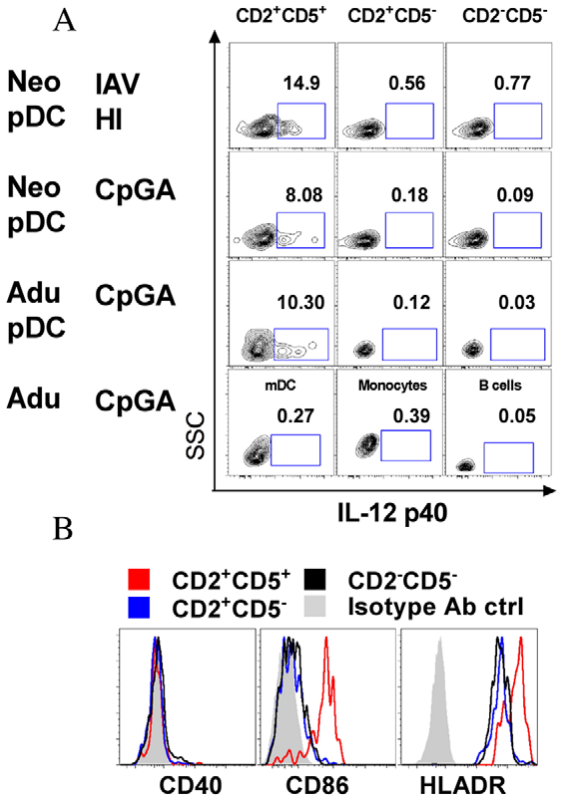
IL-12p40 producing pDCs represent a small subset of CD2+pDC. (A) Neonatal and Adult MNCs were stimulated with IAV-HI CpGA then CD2^+^CD5^+^, CD2^+^CD5^−^ and CD2^−^CD5^−^ pDCs subsets were gated to analyze their capacity to produce IL-12 p40 detected by intracellular cytokine staining. CpGA stimulated mDC (BDCA1^+^CD20^−^CD14^−^), monocytes (CD14^+^) and B cells (CD20^+^) from adult PBMC were also stained for IL-12p40 intracellularly. Numbers indicate the percentage of cytokine-producing cells among totally gated cells. (B) Expression of co-stimulatory markers CD40, CD86 and HLADR on neonatal BDCA4^+^CD123^+^pDCs defined as CD2^+^CD5^+^, CD2^+^CD5^−^ and CD2^−^CD5^−^ subsets under steady state. Data are representative of 3 experiments.

Mouse pDCs are clearly described for the production of both IFN-α and IL-12 following TLR7/9 activation by viruses and synthetic agonists. Human pDCs seem to be much more restricted in their antiviral responses by producing IFN-α but not IL-12 [22]. Nevertheless, a small fraction of mature pDC can produce IL12 but not IFN-α [23]. Recently, CD2 was highlighted as a potential marker for human pDCs subsets [24], and CD2+pDCs were shown to produce IL-12p40 in addition to other properties including IFN-α production. Importantly, the frequency of CD2+ and CD2- pDCs differed markedly between neonates and adult (Fig. 4A and B), therefore we assessed the capacity of both subsets to respond to CpGA or IAV and HIV viruses (Fig. 4C and D). Both neonatal and adult pDCs subsets similarly produced IFN-α. Non responders to CpGA was not associated with a particular subset and could respond to HIV and IAV (Fig. 4C). We further investigated IL-12p40 production by CD2+ vs. CD2- pDCs, however we identified among CD2+pDCs that only a minor population expressing CD5 did produce IL-12p40 in response to CpGA and to HI-IAV (Fig. 5A). Steady state CD5+CD2+ pDC produced much less IFN-α following TLR activation as compared to other pDC subsets (Fig. 4D). These results are in agreement with the dichotomy between IFN-α and IL-12 producing cells [23]. Interestingly, CD5+CD2+ pDC at steady state have a mature phenotype with increased CD86 and HLADR expression as compared to other pDCs (Fig. 5B) and thus probably more prone to play antigen-presenting cell functions. Again no difference was observed between neonatal and adult pDCs. IL-12p40 was not induced in myeloid DC and monocytes following CpGA activation (Fig. 5A) in accordance with the lack of TLR 9 expression in these cells [25]. IL-12p70 and IL-23 were not detected in agreement with previous studies [22]. IL-12p40 has been suggested to be a chemoattractant for macrophages in the context of bacterial infection, whether it may play a similar function in viral infection or in antigen-presenting cell related functions of pDCs remains to be clarified [26], [27]. Altogether, these results show that neonatal pDCs potently produce IFN-α in response to live and inactivated viruses. Recent publications highlighted the age dependent maturation of TLR responses [4], [28]. We provide evidence that this is not the case at the level of antiviral responses of isolated pDC and for bulk mononuclear cells for a variety of viruses. This points out the fact that age-dependent maturation may not occur at the level of innate cells, but may rather be controlled at the level of their environment to express optimal functions. Therefore, age-dependent regulatory phenomenon rather than innate deficiencies should be considered for limited inflammatory responses and susceptibility to infections seen in earlylife. We demonstrated that neonatal pDCs can potently participate in innate immunity in the context of viral infection, indicating that pDCs do not contribute to early life susceptibility to infections.

## Supporting Information

Figure S1 pDC phenotype, gating strategy and purity.
(DOCX)

Figure S2 IFN-α response to CpG by neonatal MNC or pDCs were compared according to sex, mode of delivery -cesarian (CS) or natural delivery (ND), and gestational age at birth.
(DOCX)
