# Supplementary material for: Neonatal Plasmacytoid Dendritic Cells (pDCs) Display Subset Variation but Can Elicit Potent Anti-Viral Innate Responses
Source: PLoS One. 2013 Jan 10;8(1):e52003. doi: 10.1371/journal.pone.0052003 (PMC3542339; doi:10.1371/journal.pone.0052003)

**Figure S1 :** pDC phenotype, gating startegy and purity.

The pDCs are characterized as CD123high CD45RAhigh BDCA4+ BDCA2+. Following size selection and doublets exclusion (3 left panels), only CD123high CD45RAhigh (4th panel) are found to express typically BDCA4 and BDCA2 markers as shown with blue dots (right panel). In the study for phenotypic analysis we used either BDCA2 or BDAC4. For cell purification and functional studies, BDCA 2 staining

were avoided because of reported inhibitory effect, and BDCA4 staining was not used because of competition with BDCA4 beads used for magnetic cell enrichment. Therefore, in these particular cases, FACS analysis was based on CD123high CD45RAhigh expression.

For cell purification, following Ficoll MNC were isolated (fraction A), enriched by magnetic cell sorting using BDCA4 beads (fraction B) and purified by FACS as CD123RAhigh CD45RAhigh. Purity of pDC isolated from adults (fraction C) and neonates (fraction D) were routinely superior to 99%. In some experiments, neonatal pDCs were further sorted as CD2+ (fraction E) and CD2- (fraction F) cells with high purity.


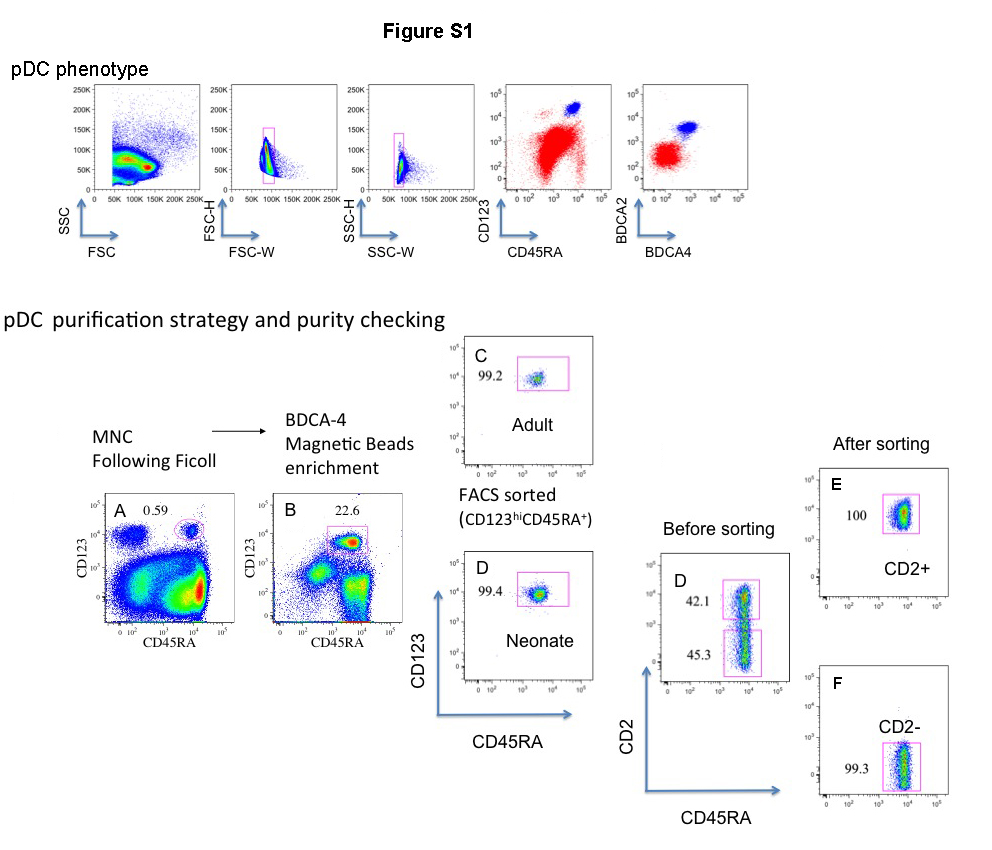

Supplement: Figure S1 — pDC phenotype, gating strategy and purity. (DOCX) [file pone.0052003.s001.docx]
