# Supplementary material for: Neonatal Plasmacytoid Dendritic Cells (pDCs) Display Subset Variation but Can Elicit Potent Anti-Viral Innate Responses
Source: PLoS One. 2013 Jan 10;8(1):e52003. doi: 10.1371/journal.pone.0052003 (PMC3542339; doi:10.1371/journal.pone.0052003)
Supplement: Figure S2 — IFN-α response to CpG by neonatal MNC or pDCs were compared according to sex, mode of delivery -cesarian (CS) or natural delivery (ND), and gestational age at birth. (DOCX) [file pone.0052003.s002.docx]

**Figure S2 :** IFN-α response to CpG by neonatal MNC or pDCs were compared according to sex, mode of delivery -cesarian (CS) or natural delivery (ND), and gestational age at birth. Number of donors are indicated in the panels.
